# Pancreatic Pseudocysts: A Retrospective Comparative Study of Contemporary Management Strategies at a Tertiary Care Centre in Central India

**DOI:** 10.7759/cureus.108946

**Published:** 2026-05-16

**Authors:** Somya Pandey, Kunwar Prativyom S Mukutawat, Krishnanand Anand

**Affiliations:** 1 Department of General Surgery, LN Medical College and Research Center, Bhopal, IND

**Keywords:** management of pancreatic pseudocyst, open cystogastrostomy, pancreas lesion, pancreatic pseudocyst, percutaneous drainage of pancreatic pseudocyst

## Abstract

Background: Pancreatic pseudocysts are common sequelae of acute and chronic pancreatitis. In India, where alcohol-related and tropical pancreatitis are prevalent, management strategies vary across institutions based on available expertise and resources. The optimal treatment approach remains debated, particularly in centres without advanced endoscopic capabilities.

Objective: To compare clinical outcomes, complications, and hospital stay duration of conservative, percutaneous, and surgical management of pancreatic pseudocysts at a tertiary care centre in central India.

Methods: This retrospective observational study included 28 consecutive patients diagnosed with pancreatic pseudocysts between January 2024 and December 2025 at JK Hospital, Bhopal. Patients were grouped based on primary management: conservative (n=8), percutaneous drainage (n=13), or surgical cystogastrostomy (n=7). Primary outcomes included resolution rate, recurrence, complications, and hospital stay.

Results: Mean patient age was 42.1 ± 13.2 years with male predominance (22, 78.6%). Alcohol consumption was the leading etiology (15, 53.6%), followed by idiopathic (seven, 25.0%) and gallstone-related pancreatitis (five, 17.9%). Conservative management achieved 75.0% resolution in selected asymptomatic patients with small pseudocysts (<6 cm). Percutaneous drainage showed initial success in 11 (84.6%) patients, but five (45.5%) had recurrence requiring surgical intervention. Surgical cystogastrostomy achieved definitive resolution in seven (100%) patients with no recurrences, median hospital stay of nine days, and one (14.3%) complication. No mortality was observed in any treatment group.

Conclusion: Management of pancreatic pseudocysts should be individualized based on cyst characteristics, symptoms, and available resources. Conservative treatment remains appropriate for asymptomatic patients with small immature pseudocysts. Percutaneous drainage provides temporary relief but has high recurrence rates. Surgical cystogastrostomy offers definitive treatment with excellent outcomes for symptomatic mature pseudocysts. In centers without advanced endoscopic capabilities, a stepwise approach from conservative to percutaneous to surgical management provides safe and effective outcomes.

## Introduction

Pancreatic pseudocysts are encapsulated collections of fluid with a well-defined inflammatory wall, typically developing four to six weeks after an episode of acute pancreatitis or as a complication of chronic pancreatitis. Unlike true cysts, pseudocysts lack an epithelial lining and are instead bounded by fibrous or granulation tissue. The reported incidence ranges from 5-16% following acute pancreatitis and affects up to 20-40% of patients with chronic pancreatitis [1,2].

In India, the epidemiology of pancreatic pseudocysts differs from Western populations due to the higher prevalence of tropical pancreatitis and alcohol-related chronic pancreatitis. Studies from tertiary care centres in India report alcohol consumption as the leading etiology (45-55%), followed by idiopathic pancreatitis (20-30%) and gallstone-related disease (15-20%). The mean age of presentation is typically younger (35-45 years) compared to Western data, with a pronounced male predominance (male: female ratio of 4:1 to 7:1) [3-5].

The natural history of pancreatic pseudocysts is variable. Approximately 40-50% of acute pseudocysts less than 6 cm in diameter may resolve spontaneously within six weeks without intervention. However, symptomatic pseudocysts or those complicated by infection, hemorrhage, or rupture require active management. Traditional surgical approaches have been progressively supplemented by minimally invasive techniques including percutaneous and endoscopic drainage in centres with advanced capabilities.

While endoscopic ultrasound (EUS)-guided drainage has revolutionized pseudocyst management in specialized centres, access to advanced endoscopic expertise and equipment remains limited in many parts of India, particularly in tier-2 and tier-3 cities. Many centres continue to rely on conservative management, percutaneous drainage, and surgical intervention. This reality necessitates evaluation of management strategies appropriate for resource-constrained settings without advanced endoscopic capabilities.

The primary objective of this study was to compare the clinical outcomes and safety profiles of conservative, percutaneous, and surgical management of pancreatic pseudocysts at JK Hospital, Bhopal, a tertiary care centre without advanced endoscopic drainage facilities. Secondary objectives included identification of factors predicting treatment success and development of an evidence-based management algorithm suitable for centres with similar resource limitations.

## Materials and methods

Study design and setting

This retrospective observational cohort study was conducted at JK Hospital, Bhopal, Madhya Pradesh. Given the retrospective nature, the requirement for individual patient consent was waived.

Patient selection

All consecutive patients diagnosed with pancreatic pseudocysts between January 2024 and December 2025, were identified through electronic medical records and hospital information systems.

Inclusion criteria were (1) age ≥18 years, (2) radiologically confirmed pancreatic pseudocyst on contrast-enhanced computed tomography (CECT) or magnetic resonance imaging (MRI), (3) minimum follow-up of six months, and (4) complete medical records.

Exclusion criteria included (1) pancreatic neoplastic cysts, (2) acute fluid collections not meeting criteria for organized pseudocysts, (3) patients transferred to other facilities before treatment completion, and (4) incomplete data precluding outcome assessment.

Of 35 patients initially identified, seven were excluded: three had neoplastic cysts, two had acute peripancreatic fluid collections, and two were lost to follow-up. The final study cohort comprised 28 patients.

Diagnostic protocol

**Figure 1:**
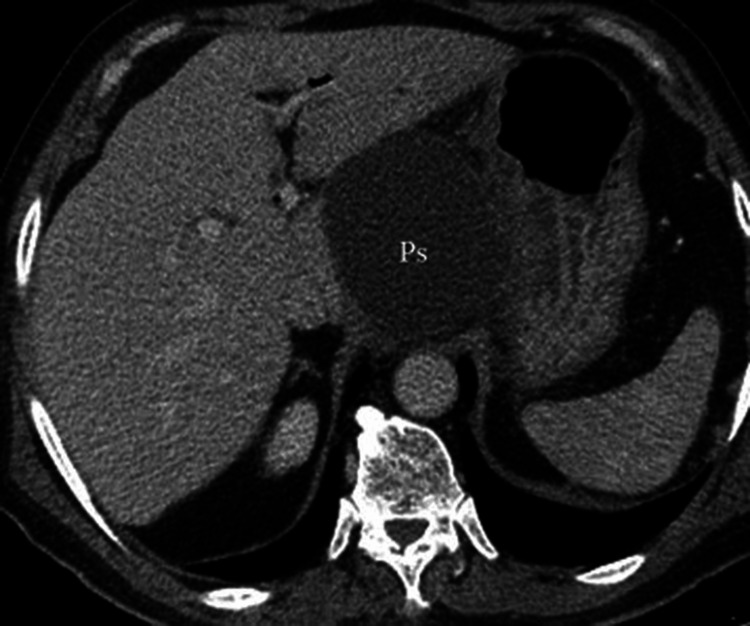
Preoperative contrast-enhanced computed tomography (CECT) abdomen (axial section) revealing a giant pancreatic pseudocyst (Ps) in the lesser sac measuring in the region of the body and tail of pancreas, with no internal debris or enhancing components, for which cystogastrostomy was planned

**Figure 2:**
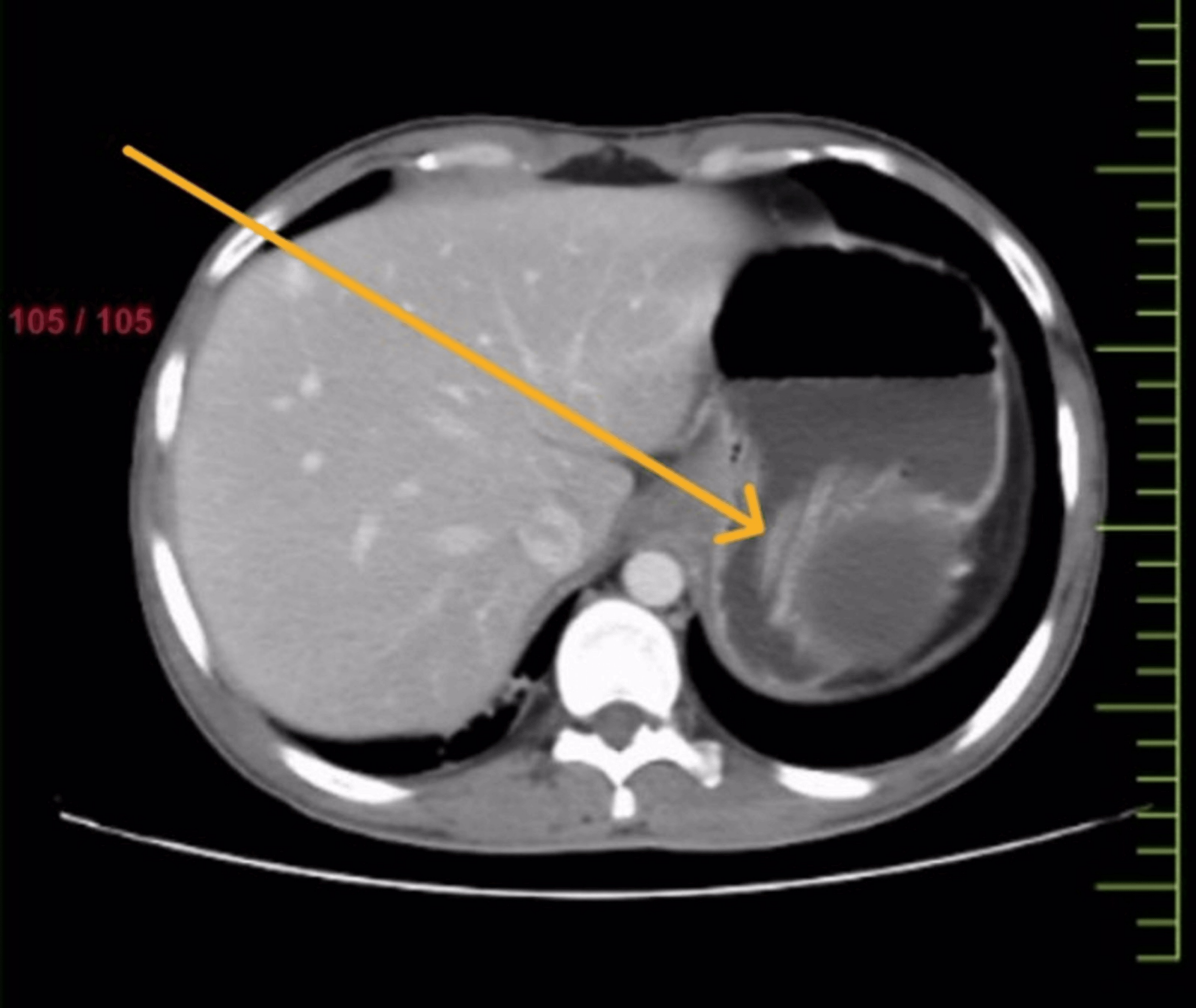
Arrow showing a well-defined, round to ovoid, fluid-attenuation collection noted in the left upper abdomen, located below the diaphragm in the region of the splenic hilum and splenic recess. The lesion abuts the adjacent gastric wall and appears closely related to the tail of the pancreas. It demonstrates a thin, smooth wall without internal septations or solid enhancing components. Findings suggestive of a pancreatic pseudocyst.

All patients underwent comprehensive clinical evaluation, including detailed history, physical examination, and laboratory investigations (complete blood count, serum amylase, lipase, liver function tests, and C-reactive protein). CECT abdomen was the primary imaging modality as seen in Figure 1 and Figure 2. Pseudocyst characteristics recorded included size (maximum diameter), location, wall thickness, communication with pancreatic duct, and presence of complications (infection, hemorrhage, or vascular involvement).

Pseudocyst maturity was defined as a well-defined wall on imaging present for at least four to six weeks from the onset of acute pancreatitis or exacerbation of chronic pancreatitis. Infected pseudocysts were diagnosed based on clinical signs of sepsis, elevated inflammatory markers, and gas within the collection on imaging or positive fluid culture.

Group 1: Conservative Management (n=8)

Criteria included asymptomatic patients with pseudocysts <6 cm, immature pseudocysts less than four weeks old, or patients initially declining intervention. Management included dietary modifications (low-fat diet), analgesics, and pancreatic enzyme supplementation when indicated. Serial imaging was performed at two, six, and 12 weeks to monitor cyst evolution.

Group 2: Percutaneous Drainage (n=13)

**Figure 3:**
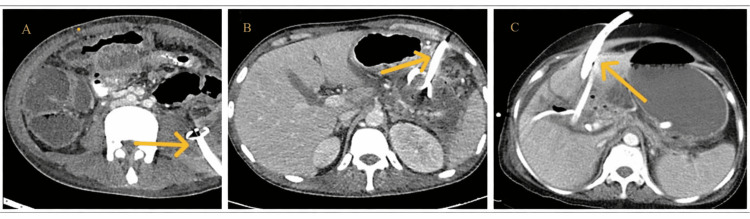
A- Retroperitoneal route of percutaneous catheter drainage of pancreatic pseudocyst, B- Transperitoneal route of percutaneous catheter drainage of pancreatic pseudocyst, C- Transhepatic route of percutaneous catheter drainage of pancreatic pseudocyst

Indicated for infected pseudocysts requiring urgent drainage, symptomatic patients who were poor surgical candidates due to comorbidities, or as a temporizing measure in acutely ill patients. Under ultrasound or CT guidance, 12-14 French pigtail catheters were inserted using Seldinger technique under local anesthesia with sedation with different approaches as depicted in Figure 3. Catheters were maintained until daily output decreased to <10 mL and follow-up imaging confirmed significant resolution or complete disappearance of the collection. Catheter care included daily flushing with normal saline and monitoring for signs of infection or dislodgement.

Group 3: Surgical Cystogastrostomy (n=7)

**Figure 4:**
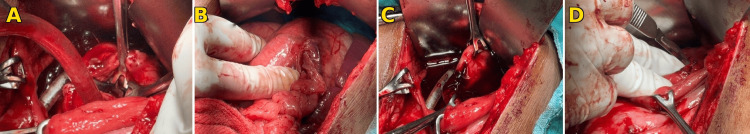
A- Exposure and identification of the pseudocyst. The large pseudocyst is visualized posterior to the stomach. Retractors hold the field open. The bluish-black discoloration of the posterior gastric wall indicates the pseudocyst bulging into it. B- Anterior gastrotomy. The anterior wall of the stomach has been opened. The surgeon's hand is seen palpating the posterior gastric wall to locate the area of maximum bulge of the pseudocyst beneath. C- Entry into the pseudocyst. The posterior gastric wall is being punctured to enter the pseudocyst cavity. Dark haemorrhagic fluid is seen draining, confirming entry into the pseudocyst. D- Opening. The communication between the stomach and pseudocyst is being enlarged to create an adequate stoma for drainage.

Reserved for symptomatic mature pseudocysts adherent to the posterior wall of the stomach, failed percutaneous drainage with recurrence, large pseudocysts (>8 cm) causing mass effect or persistent symptoms, or patient preference for definitive treatment. All procedures were performed via open anterior cystogastrostomy through an upper midline laparotomy. After identifying the pseudocyst bulge on the posterior gastric wall, a gastrotomy was made on the anterior wall, and the posterior gastric wall was incised over the maximum bulge of the pseudocyst. A generous cystogastrostomy (approximately 4-5 cm) was created, edges were secured with interrupted absorbable sutures, and the cyst cavity was thoroughly inspected and irrigated. The anterior gastrotomy was closed in two layers. Intraoperative steps are depicted in Figure 4. Nasogastric decompression was maintained for three to five days postoperatively.

Outcome measures

Primary outcomes included: (1) treatment success, defined as complete resolution of pseudocyst on imaging within 12 weeks with symptom relief; (2) recurrence rate during the follow-up period; (3) complication rate; and (4) duration of hospital stay.

Secondary outcomes comprised: (1) need for reintervention or change in management strategy, (2) total treatment cost, and (3) quality of life assessment using SF-36 questionnaire at six months post-treatment [6].

Major complications included bleeding requiring intervention, perforation, new-onset organ failure, or need for reoperation. Minor complications included transient fever, mild bleeding not requiring intervention, wound infection managed with antibiotics, or catheter-related issues managed conservatively.

## Results

Patient demographics and baseline characteristics

The study included 28 patients with a mean age of 42.1 ± 13.2 years (range: 21-68 years). There was pronounced male predominance with 22 males (78.6%) and six females (21.4%), yielding a male-to-female ratio of 3.7:1. Baseline characteristics are summarized in Table 1.

**Table 1:** Baseline Patient Demographics and Pseudocyst Characteristics SD- standard deviation cm- centimeters

Characteristic	Value	Percentage
Total patients	28	100%
Age (years), mean ± SD	42.1 ± 13.2	-
Age range (years)	21-68	-
Male gender	22	78.6%
Female gender	6	21.4%
Etiology		
Alcohol-related	15	53.6%
Idiopathic	7	25.0%
Gallstone-related	5	17.9%
Post-traumatic	1	3.6%
Type of pancreatitis		
Acute pancreatitis	18	64.3%
Chronic pancreatitis	10	35.7%
Pseudocyst location		
Head/neck	9	32.1%
Body	13	46.4%
Tail	6	21.4%
Mean size (cm)	8.4 ± 3.6	-
Size range (cm)	3.2-15.8	-
Size >8 cm	12	42.9%
Ductal communication	10	35.7%
Infected pseudocyst	4	14.3%
Symptomatic at presentation	20	71.4%

The most common etiology was alcohol-related pancreatitis (n=15, 53.6%), followed by idiopathic pancreatitis (n=7, 25.0%), gallstone-related (n=5, 17.9%), and post-traumatic (n=1, 3.6%). The majority of pseudocysts developed following acute pancreatitis (n=18, 64.3%), while 10 patients (35.7%) had underlying chronic pancreatitis.

Mean pseudocyst size was 8.4 ± 3.6 cm (range: 3.2-15.8 cm), with 12 patients (42.9%) having pseudocysts >8 cm. The most common location was the body of pancreas (n=13, 46.4%), followed by head/neck (n=9, 32.1%) and tail (n=6, 21.4%). Ductal communication was identified in 10 patients (35.7%) on MRCP or CT imaging. Four patients (14.3%) presented with infected pseudocysts.

Treatment outcomes

Treatment outcomes varied across the three management groups (Table 2). Overall treatment success rate was 85.7% (24/28 patients) when considering definitive resolution without recurrence.

**Table 2:** Treatment Outcomes Across Management Groups

Outcome Parameter	Conservative (n=8)	Percutaneous (n=13)	Surgical (n=7)
Technical success	N/A	13 (100%)	7 (100%)
Initial complete resolution	6 (75.0%)	11 (84.6%)	7 (100%)
Recurrence	0 (0%)	5 (45.5%)	0 (0%)
Definitive success	6 (75.0%)	6 (46.2%)	7 (100%)
Total complications	0 (0%)	4 (30.8%)	1 (14.3%)
Major complications (Grade III+)	0 (0%)	0 (0%)	0 (0%)
Minor complications (Grade I-II)	0 (0%)	4 (30.8%)	1 (14.3%)
Mortality	0 (0%)	0 (0%)	0 (0%)
Hospital stay (days)†	2.0 ± 0.7	7.0 (5-10)	9.0 (7-12)
Catheter/drain duration (days)	N/A	24 (16-38)	N/A
Need for subsequent intervention	2 (25.0%)	7 (53.8%)‡	0 (0%)
Mean follow-up (months)	17.8 ± 5.9	19.2 ± 7.4	21.4 ± 7.8

*Conservative Management Group (n=8)* 

Six patients (75.0%) achieved spontaneous resolution within a median of nine weeks (range: 5-14 weeks). These patients had a mean pseudocyst size of 4.8 ± 1.2 cm. Two patients (25.0%) eventually required intervention: one underwent percutaneous drainage at 8 weeks due to increasing cyst size and new-onset pain, and another underwent surgical cystogastrostomy at 12 weeks for persistent symptomatic pseudocyst. No complications occurred in patients managed conservatively. Mean follow-up in this group was 17.8 ± 5.9 months, with no late recurrences observed in those who achieved initial resolution.

*Percutaneous Drainage Group (n=13)* 

Initial technical success was achieved in all 13 patients (100%). Mean pseudocyst size in this group was 9.2 ± 3.4 cm. Eleven patients (84.6%) achieved initial complete resolution with catheter drainage. However, five of these 11 patients (45.5%) developed recurrence during follow-up (median time to recurrence: 4.8 months, range: 2-9 months). All five patients with recurrence subsequently underwent successful surgical cystogastrostomy. Overall, only six patients (46.2%) in the percutaneous group achieved definitive resolution without requiring surgical intervention.

Complications in the percutaneous group occurred in four patients (30.8%): catheter dislodgement requiring reinsertion (n=2), catheter site infection managed with antibiotics (n=1), and persistent cutaneous fistula that eventually closed spontaneously after six weeks (n=1). Median catheter duration was 24 days. Median hospital stay for the initial drainage procedure was seven days, though patients requiring subsequent surgery had additional hospitalization.

*Surgical Cystogastrostomy Group (n=7)* 

All seven surgical cystogastrostomy procedures achieved technical success with 100% resolution of pseudocysts. This included five patients who had failed percutaneous drainage (recurrence after initial success) and two patients who underwent primary surgical management due to large symptomatic pseudocysts with posterior gastric wall adherence. Mean pseudocyst size in the primary surgical subgroup was 11.4 ± 2.8 cm.

No recurrences were observed during mean follow-up of 21.4 ± 7.8 months. Complications occurred in one patient (14.3%): surgical site infection requiring prolonged antibiotic therapy and delayed wound healing. This patient eventually achieved complete recovery. No patients experienced major complications such as bleeding requiring intervention, anastomotic leak, or need for reoperation. Median hospital stay was nine days. All patients resumed normal diet within five to seven days postoperatively.

No mortality was observed in any of the three treatment groups during the study period or follow-up.

Factors influencing treatment success

Analysis of factors influencing treatment success revealed several clinically relevant patterns (Table 3). Pseudocyst size emerged as the most important predictor: patients with pseudocysts <6 cm had 90.0% success with conservative management (9/10 patients), while those with pseudocysts >8 cm had only 16.7% success with conservative approach (2/12 patients).

**Table 3:** Factors Associated with Treatment Success PCD- Percutaneous drainage

Factor	Success Rate	Clinical Observation
Pseudocyst size <6 cm	9/10 (90.0%)	Excellent for conservative Rx
Pseudocyst size >8 cm	2/12 (16.7%)	Poor for conservative Rx
Ductal communication present	3/10 (30.0%)	High PCD recurrence rate
Ductal communication absent	16/18 (88.9%)	Better outcomes all modalities
Infected pseudocyst	3/4 (75.0%)	Required urgent PCD
Wall maturity >6 weeks	7/7 (100%)	Essential for surgery
Chronic pancreatitis	4/10 (40.0%)	Higher PCD recurrence
Acute pancreatitis	18/18 (100%)	Better overall outcomes

Communication with the main pancreatic duct significantly reduced success in percutaneous drainage: only three of seven patients (42.9%) with ductal communication achieved definitive resolution with percutaneous drainage alone, compared to three of six patients (50.0%) without ductal communication. All patients with ductal communication who underwent surgical cystogastrostomy achieved successful outcomes.

Infected pseudocysts (n=4) had poor outcomes with conservative management (0/1) but responded well to percutaneous drainage for initial source control (3/3), though two of these later required surgical intervention for recurrence. Wall maturity over six weeks was present in all surgical candidates and was associated with successful anastomosis formation without leakage.

Patients with chronic pancreatitis (n=10) had a higher tendency toward recurrence after percutaneous drainage (60%, 3/5) compared to those with acute pancreatitis etiology (25%, 2/8). However, surgical outcomes were uniformly successful regardless of underlying pancreatitis type.

## Discussion

This retrospective study of 28 patients with pancreatic pseudocysts represents our institutional experience comparing management strategies available at JK Hospital, Bhopal, a tertiary care centre without advanced endoscopic drainage capabilities. Our findings demonstrate that excellent outcomes can be achieved using a stepwise approach incorporating conservative management, percutaneous drainage, and surgical cystogastrostomy, even in the absence of therapeutic endoscopy.

Demographic and etiological patterns

The demographic profile in our cohort reflects typical patterns seen across Indian tertiary care centres. The mean age of 42.1 years and strong male predominance (78.6%) with a male-to-female ratio of 3.7:1 are consistent with published Indian data. Alcohol-related pancreatitis as the leading etiology (53.6%) aligns with national trends showing alcohol as the predominant cause of pancreatitis-related complications in India.

The relatively high proportion of idiopathic cases (25.0%) may represent undiagnosed tropical pancreatitis (more common in southern India but occasionally seen in central regions), genetic mutations not routinely tested for, or under-reporting of alcohol consumption due to social stigma. The lower incidence of gallstone-related pseudocysts (17.9%) compared to Western series reflects the younger patient population and different risk factor profile in our setting.

Conservative management

Our success rate of 75.0% with conservative management in carefully selected patients validates the strategy of initial observation for small, asymptomatic pseudocysts. This aligns with international guidelines recommending conservative approaches for uncomplicated pseudocysts, particularly those <6 cm in diameter [7,8]. The mean resolution time of nine weeks is consistent with the natural history of acute pseudocysts [9].

Important predictors of successful conservative management in our series included: pseudocyst size <6 cm (90% success rate), absence of symptoms at presentation, lack of ductal communication, and acute rather than chronic pancreatitis etiology. The 25% failure rate requiring subsequent intervention emphasizes the need for close monitoring with serial imaging and clear patient counselling about warning signs requiring urgent evaluation.

Percutaneous drainage

While percutaneous drainage achieved high initial technical success (100%) and initial resolution (84.6%), the significant recurrence rate (45.5%) limits its role as definitive treatment. These results are consistent with published literature showing recurrence rates of 20-50% [10,11] and highlight a fundamental limitation of external drainage: it does not create a permanent internal drainage pathway.

Despite this limitation, percutaneous drainage maintains important roles in our practice: (1) emergency decompression of infected pseudocysts (all three infected cases in this group responded well to initial drainage), (2) temporizing measure in critically ill or nutritionally depleted patients to allow optimization before definitive surgery, (3) management of poor surgical candidates with significant comorbidities who may tolerate repeated drainage better than major surgery, and (4) diagnostic tool when malignancy cannot be excluded (fluid cytology and tumor markers).

The high recurrence rate, particularly in patients with ductal communication (57% vs. 33% without communication) and chronic pancreatitis (60% vs. 25% in acute pancreatitis), suggests that percutaneous drainage should be viewed as a bridge to surgery in many cases rather than definitive treatment [12]. The need for prolonged catheter maintenance (median 24 days) and associated complications (dislodgement, infection, persistent fistula) add to patient burden and healthcare costs.

Surgical cystogastrostomy

Surgical cystogastrostomy emerged as the most definitive treatment modality in our study, achieving 100% resolution with zero recurrence during a mean follow-up exceeding 21 months. These excellent outcomes validate the role of surgery as the gold standard for definitive management of pancreatic pseudocysts, particularly in centres without advanced endoscopic capabilities.

The low complication rate (14.3%, one surgical site infection) in our series, with no major complications such as bleeding, anastomotic leak, or need for reoperation, compares favourably with published surgical series [13,14].

Our preference for anterior cystogastrostomy through the posterior gastric wall is based on several advantages: (1) direct access to pseudocysts adherent to the stomach, (2) creation of a large, permanent internal drainage pathway (4-5 cm anastomosis), (3) ability to directly inspect and irrigate the cyst cavity, (4) elimination of external drainage with associated fistula risk, and (5) technical simplicity compared to cystojejunostomy.

The median hospital stay of 9 days, while longer than reported for endoscopic drainage (typically two to four days) [15,16], is acceptable given the definitive nature of the procedure and zero recurrence rate. Early mobilization, prompt resumption of oral feeding (five to seven days), and standardized postoperative care pathways have helped minimize hospital stay while ensuring safety.

Management in resource-limited settings

Our experience demonstrates that centres without advanced endoscopic drainage facilities can achieve excellent outcomes using a rational, stepwise approach. The key is appropriate patient selection for each modality based on cyst characteristics, symptoms, and patient factors.

We propose the following algorithm based on our experience: (1) Conservative management for asymptomatic pseudocysts <6 cm, particularly in acute pancreatitis, with observation for six to eight weeks; (2) Percutaneous drainage for infected pseudocysts requiring urgent decompression, or as temporizing measure in critically ill patients; (3) Surgical cystogastrostomy for symptomatic mature pseudocysts adherent to the stomach, failed conservative management, failed or recurrent percutaneous drainage, or large pseudocysts (>8 cm) causing mass effect.

This algorithm recognizes that while endoscopic drainage may offer advantages in terms of hospital stay and immediate recovery, surgical cystogastrostomy provides definitive treatment with excellent outcomes in experienced hands. For many patients in resource-limited settings, a well-executed surgical drainage procedure with zero recurrence risk may be preferable to multiple endoscopic procedures or repeated percutaneous interventions.

## Conclusions

Management of pancreatic pseudocysts requires individualized treatment based on clinical presentation, cyst characteristics, and available institutional resources. Our experience at JK Hospital demonstrates that excellent outcomes can be achieved even without advanced endoscopic drainage capabilities by employing a rational, stepwise approach. Conservative management remains appropriate for selected asymptomatic patients with small, immature pseudocysts, achieving 75% success rate with careful monitoring. Percutaneous drainage serves important roles in managing infected pseudocysts and critically ill patients but should be recognized as a bridge to definitive therapy rather than definitive treatment itself, given the recurrence rate in our series. Surgical cystogastrostomy provides definitive treatment with 100% success rate, zero recurrence, and acceptable morbidity in our series. For symptomatic mature pseudocysts adherent to the stomach, particularly in centers without therapeutic endoscopy capabilities, surgical drainage represents the gold standard approach. In resource-limited settings, a stepwise algorithm prioritizing conservative management for appropriate cases, judicious use of percutaneous drainage for specific indications, and definitive surgical cystogastrostomy for symptomatic mature pseudocysts can achieve outcomes comparable to centres with advanced endoscopic capabilities. The key to success lies in appropriate patient selection, multidisciplinary collaboration, and surgical expertise in performing drainage procedures.
